# Covid-19 Kills More Men Than Women: An Overview of Possible Reasons

**DOI:** 10.3389/fcvm.2020.00131

**Published:** 2020-07-17

**Authors:** Annalisa Capuano, Francesco Rossi, Giuseppe Paolisso

**Affiliations:** ^1^Department of Experimental Medicine, University of Campania Luigi Vanvitelli, Regional Centre of Pharmacovigilance, Campania Region, Naples, Italy; ^2^Department of Advanced Medical and Surgical Sciences, University of Campania Luigi Vanvitelli, Naples, Italy

**Keywords:** Covid-19, mortality, gender difference, immune system, sex hormones, smoking habit, coagulation pattern, cardiovascular diseases

## Abstract

The high mortality observed in Covid-19 patients may be related to unrecognized pulmonary embolism, pulmonary thrombosis, or other underlying cardiovascular diseases. Recent data have highlighted that the mortality rate of Covid-19 seems to be higher in male patients compared to females. In this paper, we have analyzed possible factors that may underline this sex difference in terms of activity of the immune system and its modulation by sex hormones, coagulation pattern, and preexisting cardiovascular diseases as well as effects deriving from smoking and drinking habits. Future studies are needed to evaluate the effects of sex differences on the prevalence of infections, including Covid-19, its outcome, and the responses to antiviral treatments.

## Introduction

All countries around the world are facing the COVID-19 emergency. As of June 22nd, more than 9,118,000 people have contracted the disease, and deaths have exceeded 471,000^1^.

From a pathogenetic point of view, the progression of COVID-19 follows three main stages (1). The first stage, which approximately occurs in the initial 1–2 days, represents the phase in which the SARS-CoV-2 binds to epithelial cells and starts replicating. The human angiotensin-converting enzyme 2 (ACE2) receptor and TMPRSS2 are the main proteins involved in the cell entry of SARS-CoV-2 (2, 3). This phase is asymptomatic, and the innate immune response is limited. The second stage starts once the virus migrates down the respiratory tract. This phase is symptomatic with clear airway response and the innate immune response is triggered. An increase in the level of CXCL10 or other innate response cytokine is observed (4, 5). Indeed, this is the stage in which the so-called “cytokine storm” arises. Lastly, about 20% of patients with COVID-19 progress to a third stage, which is the most severe, and this stage is characterized by serious respiratory symptoms that include hypoxia, ground glass infiltrate, and progression to acute respiratory distress syndrome (ARDS). This stage can be further aggravated by organ failure and sepsis, potentially progressing to patient's death (6). At this stage, an aggressive immunomodulatory therapy is probably needed to prevent the onset of serious clinical consequences, such as the Disseminated intravascular coagulation (DIC) and the subsequent consumption coagulopathy (7). Indeed, as recently reported by a group of Italian researchers, the high mortality observed among Covid-19 patients may be somewhat due to unrecognized pulmonary embolism and pulmonary *in situ* thrombosis. Therefore, they suggested that a better understanding of Covid-19-related thromboembolic risk would help to optimize diagnostic strategies but also the proper pharmacological management of patients with Covid-19 (8).

Data shared by Global Health 50/50, an internationally selected company that promotes gender equality in healthcare, revealed a higher proportion of deaths for Covid-19 in men than women in almost all countries. In Italy, according to data reported in the bulletin of integrated surveillance (update of April 23rd, 2020), deaths in men are approximately double compared to that of women (17.1 vs. 9.3%). Similar findings were reported in Greece, Holland, Denmark, Belgium, Spain, China, and the Philippines^2^. A study carried out by Liu et al. on 4,880 patients with respiratory symptoms or close contact with Covid-19 patients in a hospital in Wuhan showed that there was a significant higher rate in positivity to SARS-CoV-2 in males and the elderly population (>70 years), although only age was recognized as a risk factor (9). Similarly, a recent retrospective observational study showed that among critically ill patients with SARS-CoV-2, 67% were males and that the mortality rate was higher in males (10). In addition, a review of data related to 1,099 patients with Covid-19 showed that 58.1% were males. Furthermore, out of 173 severe cases, 57.8% concerned this population too (11). In addition, recent published data from a survival analysis (12) showed that men had a significantly higher mortality and exhibited worse symptoms than women. Lastly, Scully et al. recently reported that the case fatality rate for males is 1.7 times higher than for females (*P* < 0.0001) (13).

Considering that sex differences are frequently observed in many diseases, responses to drugs and the occurrence of adverse drugs reactions (14–17)—and that many reasons may underline these differences—in this paper, we aim to provide an overview of factors, including those influencing the immune system response, that possibly underline the sex and gender differences observed in Covid-19 patients. All those factors are summarized in Table 1.

**Table 1:** Overview of sex- and gender-differences that could be responsible of increased mortality rate in men with Covid-19.

Activity of the immune system	▘ Female patients seem to have an intense and prolonged innate, humoral, and cell-mediated immune response, leading to a faster and higher recognition of viral components ▘ Preclinical studies showed that females might recover to a greater extent and are better protected from death during infections
Role of sex hormones	▘ Testosterone shows suppressive effect on the immune function, while estrogen may have both suppressive and not suppressive effects depending on their levels ▘ In men androgens deficiency is associated with increased levels of inflammatory cytokines and increased CD4+/CD8+ T-cell ratio ▘ Estrogens are able to induce an upregulation in the expression of ACE2 ▘ Exogenous estrogen increases the clotting risk in women and in biological males undergoing gender-affirming hormonal therapy ▘ Sex hormones could also affect the response to antiviral treatments or vaccines
Prevalence of cardiovascular diseases	▘ Women seem to have a higher risk and incidence of symptomatic supraventricular tachycardia and long QT syndrome compared with men ▘ Men show higher risk of atrial fibrillation and sudden cardiac death and they are more affected by atherosclerotic cardiovascular disease compared with women
Coagulation pattern	▘ Men have a 3.6-fold higher risk of recurrent VTE than women ▘ Women show higher risk of VTE during fertile years
Smoking and drinking habits	▘ Smoking habit is higher in men than women ▘ Drinking habit is higher in men than women

## Sex Differences in Immune System

Many studies, both preclinical and clinical, have analyzed the role of the sex in immune response patterns during viral infections. Few studies have proposed that the sex variability in the prevalence, pathogenesis, and response to viral infections can be related to the greater humoral and cell-mediated immune responses of females to viral antigens (18–20). This variability is probably the driver of a lower intensity and prevalence of viral infections in females than males. Indeed, female patients seem to be less susceptible to viral infections due to intense and prolonged innate, humoral, and cell-mediated immune responses. The higher activity of innate immune system in women, which is mediated by Toll-like receptors, retinoic acid-inducible gene I-like receptors, and nucleotide oligomerization domain-like receptors, may lead to a faster and higher recognition of viral components and consequently higher production of type 1 interferon (IFN) and inflammatory cytokines (IL-1, TNFs) (21). For instance, in the United States, the 1918 influenza pandemic was associated with a higher mortality rate in men than women (22). Male gender could also be associated with higher mortality rate in herpes simplex virus-1 (HSV-1) respiratory infection. Indeed, Brown et al. evaluated the effects of sex on susceptibility to HSV-1 respiratory infection after repeated exhaustive exercise (treadmill running at 36 m/min) in CD-1 mice (86 males and 89 females). The results showed that the exercise stress was associated with increased morbidity and mortality in male mice, while only an increase in morbidity was observed in females. Authors suggested that females might recover to a greater extent and are ultimately better protected from death (23). Similar findings were found by another preclinical study carried out by Han et al. (24), while no sex differences were found for ocular HSV-1 infection (25).

On the other hand, the higher immune responses observed in females may lead to increased development of symptoms of infection in this population (26). For instance, with regard to influenza A viruses, even though men seemed to be more exposed to this infection, women showed higher mortality rates (27). During 2009 H1N1, a higher hospitalization rate was observed in young women (28). Furthermore, females had a 2-fold higher risk of death than males (29, 30). This could be the consequence of the higher immune response that leads to high levels of pro-inflammatory cytokines, including IL-1, IL-2, IL-6, G-CSF, IP-10, and TNFα, a condition that is defined as a “cytokine storm” and that seems to worsen symptoms of Covid-19 infections, such as ARDS, organ failure, and sepsis (31–33).

### The Role of Sex Hormones

Apart from factors merely related to a higher/lower activity of innate, humoral, and cell-mediated immune responses and to the production of inflammatory cytokines, other factors, including sex hormones, may play a key role during response to viral infections (34). In women, the level of estrogen varies during the menstrual cycle and falls with menopause, while, in men, the level of testosterone remains stable up to 60 years of age. Sex hormones induce their effects through the binding with estrogen receptors (ERα and ERβ), the androgen receptor (AR), and progesterone receptors (PR-A and PR-B). Innate immune cells express those receptors to varying degrees (35). Some studies have demonstrated that testosterone exhibits a suppressive effect on the immune function, while estrogen may have both suppressive or not suppressive effects depending on their levels (36–38). Data from studies carried out in humans revealed that in men androgens deficiency is associated with increased levels of inflammatory cytokines and increased CD4+/CD8+ T-cell ratio compared to men with normal level of testosterone (39, 40). On the other hand, estrogens could affect several activities of the innate and adaptive immune responses, showing opposite effects on the immune system based on their level. Indeed, low doses of estrogens seem to induce monocyte differentiation into inflammatory DCs, leading to higher production of IL-4 and IFN-α and activate Th1-type and cell-mediated immune responses. On the contrary, high doses of estrogens show inhibitory activity on innate and pro-inflammatory immune responses and enhance Th2-type responses and humoral immune responses (36, 41). Given the multiple effects of female hormones on immune system functions, women may present different responses to viral infections during the course of their lives. For instance, during pregnancy, which represents a unique immunological state, women seem to undergo three different stages: an initial pro-inflammatory phase, a second one (corresponding to the second trimester of pregnancy), which is characterized by an anti-inflammatory state, and a third phase that is characterized by an increase of inflammatory processes, which are useful for uterine muscle contraction, for the delivery as well as for placenta rejection. The succession of these pro- and anti-inflammatory phases seems to be the results of T helper 1 (Th1)/T helper 2 (Th2) immune shifts that, in turn, could also reflect a change in sensitivity to infectious diseases among pregnant women (42). Indeed, the higher mortality rate of 2009 H1N1 in women was found for those in reproductive age (20–49 years), suggesting a role of gonadal hormones, especially during pregnancy (28). Lastly, sex hormones could also affect the response to antiviral treatments or vaccines. As reported by Klein (26), the efficacy of the HSV-2 vaccine and of the recombinant glycoprotein D (gD)-based HSV-2 vaccine against the development of symptoms associated with genital herpes was found to be higher in women than in men.

## Sex Difference in Cardiovascular Diseases

Recent literature data showed a higher prevalence of hypertension and coronary artery disease in patients with severe forms of Covid-19 (43, 44), suggesting that preexisting cardiovascular diseases may lead to a worse prognosis. According to Wu et al. (45), an arrhythmogenic effect of Covid-19, with occurrence of long and short QT syndrome, Brugada syndrome, and catecholaminergic polymorphic ventricular tachycardia, could be expected (46). These life-threatening cardiac disorders can be the consequence of enhanced inflammation, which can increase the duration of ventricular repolarization, by affecting the QTc interval (47). On the other hand, heart injury can be induced by other mechanisms, including some deriving from the effects of ACE2 that is expressed in the lungs and in the cardiovascular system, while other deriving from the cytokine storm and hypoxaemia that results in myocardial cells damage^3^. Indeed, inflammatory cytokines, especially interleukin-6, increase the risk of QT interval prolongation and life-threatening arrhythmias (48). In addition, cytokines show a pro-atherogenic effect, including TNF-α, which activates NF-κB, p38 MAPK, and the transcription of proinflammatory genes for cytokines involved in the cytokine storm (49). Therefore, the role of cytokines in worsening the cardiovascular homeostasis of patients with Covid-19 cannot be excluded.

Some sex differences have been found in the incidence of cardiovascular diseases. Indeed, while women seem to have a higher risk and incidence of symptomatic supraventricular tachycardia and long QT syndrome, men show higher risk of atrial fibrillation and sudden cardiac death. Furthermore, epidemiological studies demonstrated that men are more affected by atherosclerotic cardiovascular disease compared with women. This difference can be imputable to the clinical risk profile, effects of sex hormones, and social attitude (50). Apart from sex differences in the production of inflammatory cytokines and in the incidence of cardiovascular diseases, recent evidence shows that a sex difference in virus-targeted mechanism could be hypothesized. As previously reported, the ACE2 receptor is essential for the cell entry of SARS-CoV-2, but it also represents an important enzyme of the renin-angiotensin system (RAS) that provides protective effects in many chronic conditions, like hypertension, cardiovascular diseases, and acute respiratory distress syndrome. All these clinical conditions represent risk factors for a worse prognosis in Covid-19 patients. Ruggieri and Gagliardi have recently reported that estrogens are able to induce an upregulation in the expression of ACE2, which could explain better outcome and lower death rate in women compared to men^4^. Furthermore, as recently reported by Gagliardi et al., the gene that encodes for ACE2 is on the X chromosome, and XX cells over-express it (51). Furthermore, the results of a preclinical study that investigated the role of ACE2 in angiotensin (1–7)-induced hypertension and regulation of the RAS system in the kidney of wild type and Ace2 knockout mice revealed some sex differences in rising of mean arterial pressure, binding of glomerular AT1 receptor, and renal protein expression of the neutral endopeptidase neprilysin, suggesting that females may be protected from angiotensin (1–7)-induced hypertension (52).

Even though the ACE2 plays an essential role in the RAS system, it should be highlighted that a recent retrospective cohort study, carried out on 4,480 patients with Covid-19, showed that the prior use of ACE inhibitors or angiotensin receptor blockers (both acting on RAS) was not significantly associated with COVID-19 diagnosis neither with mortality or severe disease (53).

## Sex Difference in Coagulation Pattern

The DIC is a life-threatening syndrome which leads to disseminated and uncontrolled activation of coagulation, thrombosis, and progressive consumption coagulopathy, leading to an increased bleeding risk. The DIC occurs frequently in almost 30–50% of patients with sepsis and 10% in patients with solid tumors, trauma, or obstetric calamities. Furthermore, the risk of DIC is higher in critically ill patients hospitalized in ICU, for whom the prevalence of DIC is about 8.5–34% (54). According to Tang et al., ~71.4% of the non-survivor patients with Covid-19 matched the grade of overt-DIC (≥5 points) in later stages of SARS-Cov-2 pneumonia, and 76% of the non-survivors were males. On the contrary, only the 0.6% of survivors matched the DIC criteria during the hospital stay (55). Moreover, the DIC appears to be a driver of disease severity. As might be expected, it is a strong prognostic factor for poor outcome (55). Finally, *microthrombi* have been reported as autopsy findings in patients with Covid-19^5^.

It is widely demonstrated that differential risks in men and women for cardiovascular disease exist, especially during premenopausal period due to female sex regulating hormones. Moreover, once reproductive risk factors are taken into account, men have a 3.6-fold higher risk of recurrent venous thromboembolism (VTE) than women (56). The pathophysiology behind this observation is unclear (57). Indeed, it is known that deficient coagulation problems, such as hemophilia, are under genetic control and sex-related, so one cannot exclude that hypercoagulability might be also affected by genetic factors (58). Furthermore, women show higher risk of VTE during fertile years, mainly as consequence of the effects mediated by pregnancy and oral contraceptive use. In this regard, literature data suggested that exogenous estrogen increases the clotting risk in women and in biological males undergoing gender-affirming hormonal therapy (59, 60).

## Gender Differences in Smoking and Drinking Habits

Compared to non-smokers, smokers generally show higher rates of respiratory diseases, including colds, influenza, bacterial pneumonia, and tuberculosis (61–64). Indeed, smoking habit leads to progressive lung damage, which exposes patients to higher risk of pulmonary bacterial and viral infections (65). This leads to higher risk of hospitalization due to influenza infection as well (66). Lastly, smoking represents the fourth leading cause of death in the world (67). In the context of Covid-19, smokers are more likely to contract the disease since the act of smoking implies that possibly contaminated fingers are in contact with lips, increasing the possibility of the SARS-CoV-2 virus being transmitted from hand to mouth (68, 69). Furthermore, smoking is also related to higher expression of ACE2, which is involved in the process of cell entry of the SARS-CoV-2 (70).

Generally, the percent of smoking habits is found to be higher in men than women, since the adolescent age, even though with differences among low- medium- and high-income countries (71, 72). In order to evaluate the association between smoking and Covid-19 outcomes, in terms of disease severity, need for mechanical ventilation or intensive care unit (ICU), hospitalization, and death, Vardavas et al. carried out a systematic review of studies on Covid-19 patients that included information on patients' smoking status. Authors highlighted that there were higher percentages of current and former smokers among patients who accessed to ICU, required mechanical ventilation, or who had died (73). Other studies are strongly needed to evaluate the prevalence of smokers among patients with severe Covid-19, but based on current knowledge it is possible to assume that smokers are likely to be at higher risk for severe SARS-CoV-2 infection. Therefore, smoking cessation awareness should be strongly encouraged in order to reduce the global impact of COVID-19 (74).

As for smoking habits, drinking is found to be higher in men than women. Indeed, women generally drink less and have a lower prevalence of drink problems than men (75). Alcohol-related liver disease represents one of the main causes of liver cirrhosis, associated with high mortality and morbidity. A recent study, which has analyzed the prevalence, severity and mortality of patients diagnosed with COVID-19 with underlying chronic liver diseases, showed that this disease is associated to higher severity and mortality also in Covid-19 patients^6^.

## Conclusion

Covid-19 still represents a worldwide health emergency. In this paper, we have analyzed possible factors that may have contributed to a gender difference in Covid-19 clinical outcomes, especially in the rate of death. Among possible factors, those related to the activity of immune system and the role of sex hormones seem to be the most important. However, in our opinion, sex differences in cardiovascular diseases and coagulation patterns should be considered as well, especially considering the possible role of the cytokine storm in inducing vascular inflammation and atherosclerosis-related cardiovascular diseases but also gender differences in coagulation, which can be responsible of higher risk of thrombotic/thromboembolic phenomena in men compared to women. Further epidemiological studies will be needed to confirm this. Lastly, considering that women are often underrepresented in randomized clinical trials, future studies are needed to evaluate the effects of sex differences on the prevalence of infections, their outcome, and responses to antiviral treatments.

## Author Contributions

AC, FR, and GP drafted the work and revised it for important intellectual content, made substantial contributions to the acquisition, analysis, or interpretation of data for the work, approved the final version of the manuscript, developed the concept, and wrote the manuscript. All authors contributed to the article and approved the submitted version.

## Conflict of Interest

The authors declare that the research was conducted in the absence of any commercial or financial relationships that could be construed as a potential conflict of interest.

## Footnotes

^1^https://www.worldometers.info/coronavirus/ (accessed May 10, 2020).

^2^https://www.epicentro.iss.it/coronavirus/sars-cov-2-differenze-genere-importanza-dati-disaggregati

^3^https://www.nature.com/articles/s41569-020-0360-5#citeas

^4^https://www.gendermedjournal.it/articoli.php?archivio=yes&vol_id=3351&id=33219

^5^https://www.preprints.org/manuscript/202002.0407/v2

^6^https://www.mdpi.com/2414-6366/5/2/80/htm
